# Azidohomoalanine (AHA) Metabolic Labeling Reveals Unique Proteomic Insights into Protein Synthesis and Degradation in Response to Bortezomib Treatment

**DOI:** 10.3390/proteomes13040063

**Published:** 2025-11-25

**Authors:** Lina Alhourani, Yasser Tabana, Ashwin Anand, Richard P. Fahlman

**Affiliations:** 1 Department of Biochemistry, Faculty of Medicine & Dentistry, University of Alberta, Edmonton, AB T6G 2H7, Canada; alhouran@ualberta.ca (L.A.); anand5@ualberta.ca (A.A.); 2 Department of Physiology and Biochemistry, Faculty of Medicine, Jordan University of Science and Technology, Irbid 22110, Jordan

**Keywords:** proteomics, azidohomoalanine, proteasome, proteostasis, protein degradation, protein synthesis

## Abstract

Background: Multiple myeloma (MM) is essentially an incurable cancer, but treatments with proteasome inhibitors are widely used clinically to extend patient survival. While the mechanisms of proteasome inhibition by Bortezomib are well known, the cellular responses to this proteotoxic stress that leads to sensitivity by MM are not fully elucidated. This study reports on the application of an emerging method to investigate proteostasis by proteomics. Methods: We utilized metabolic labeling with azidohomoalanine (AHA) in a MM cell line in combination with Bortezomib treatment. AHA labeling facilitates the selective isolation and identification of proteins for investigations of protein synthesis or protein degradation. Results: The data collected reveals significant changes in gene protein synthesis upon Bortezomib treatment, including protein neddylation. The data also reveals a global increase in protein degradation, which suggests the induction of an autophagy-related process. The resulting data collected reveals significant changes upon Bortezomib treatment in protein synthesis of genes, including protein neddylation, and protein degradation data reveals a global increase in protein degradation, suggesting an induction of an autophagy-related process. Subsequent cellular and proteomic analysis investigated the additional treatment of an autophagy inhibitor, hydroxychloroquine, in combination with Bortezomib treatment by label-free proteomics to further characterize the proteome-wide changes in these two proteotoxic stresses. Conclusions: AHA metabolic labeling proteomics to investigate protein synthesis and degradation enables novel complementary insights into complex cellular responses compared to that of traditional label-free proteomics.

## 1. Introduction

Multiple myeloma (MM) is the second-most frequently diagnosed hematological cancer and represents approximately 1% of all malignancies and 15% of hematological cancers [1]. Despite advancements in therapies, multiple myeloma remains an incurable malignancy, with unclear mechanisms underlying the disease’s refractory nature after drug treatment [2]. One current therapy for MM is the application of the proteasome inhibitor Bortezomib (PS-341 or Velcade), which can lead to disease remission, but eventually, a resistant form of the disease returns [3].

While Bortezomib’s molecular target and mechanism is known [4], the cellular responses that lead to the sensitivity of MM are complex and only partially understood [5,6]. Reported processes include and are not limited to the endoplasmic reticulum (ER) stress [7], nuclear factor κB signaling [8], and cell cycle inhibition [9]. For refractory disease, the understanding of drug resistance has been even more challenging with no common insights having been identified to date [5].

To obtain deeper molecular insight into the alterations to the cellular proteome upon Bortezomib treatment, there have been many reports using traditional proteomics in this system. Recent reports have been insightful in revealing the roles of the unfolded protein response [10] and potential applications in drug-sensitivity screening [11]. Here, we report on the MM cellular response to Bortezomib using emerging proteomic applications that can selectively monitor either protein synthesis or protein degradation. The application of these parallel methods using the methionine analog azidohomoalanine in metabolic labeling experiments provides novel insights into cellular proteostasis of a MM model that have not been previously characterized by traditional proteomic methods.

## 2. Materials and Methods

### 2.1. Cell Culture

RPMI-8226 cells were obtained from Dr. Raymond (University of Alberta, Canada) and used to study the effects of Bortezomib. They were maintained in RPMI supplemented with 10% Fetal bovine serum (FBS) (SIGMA#F1051) and 1× penicillin. In total, 5 × 10^5^ cells/mL were grown in 32 untreated dishes.

### 2.2. Chemicals

Bortezomib was purchased from MCE (Monmouth Junction, NJ, USA), Cat. #HY-10227; hydroxychloroquine from Thermo Fisher Scientific (Waltham, MA, USA), lot #A010025101.

### 2.3. Cell Viability Assays

CCK-8 assays were performed to assess the cell viability. In brief, RPMI-8226 cells were cultured onto 96-well plates at a density of 5 × 10^3^ cells per well at serial concentration gradients of Bortezomib, with three repeats for each concentration. Cell-counting Kit-8 (DOJINDO (Rockville, MD, USA) #CK04) reagent was used as recommended by the supplier to quantify cell viability. Cell counting using the trypan blue exclusion test was performed manually.

### 2.4. Azidohomoalanine (AHA) Pulse–Chase Method

The RPMI-8226 cells were grown in 10 cm dishes to reach a density of 500,000 cells/mL before starting the pulse–chase experiment. At the start, the cells were washed 2× with methionine-free (Met-) RPMI (Thermo Fisher Scientific, #A1451701) to eliminate any excess Met. Subsequently, the live labeling was performed for 16 h, utilizing 50 μM of L-azidohomoalanine (AHA) (Vector Laboratories (Newark, CA, USA) #CCT-1066) in Met-RPMI, with 10% dialyzed FBS (Cytiva (Marlborough, MA, USA) #SH30079.01). A total of 0.2 mM cysteine (Sigma-Aldrich (Burlington, MA, USA) #8755) and 2 mM L-glutamine (GIBCOBRL (Life Technologies Corp, Carlsbad, CA, USA) #21051-016) were added. The control cells were cultured at the same time in a regular RPMI medium containing (Met+) and 10% (FBS). Then, the cells were rinsed 2× with Met+ RPMI and then kept in regular media for 0–8 h at 37 °C (chase phase). Cells were lysed either immediately following the pulse (control and 0 h) or at various time intervals during the chase periods (2, 4, and 8 h). The lysis buffer was 1% SDS in PBS, which included a protease inhibitor cocktail tablet from Complete Roche that did not contain EDTA (Thermo Fisher Scientific, Cat#1873580). Afterward, the lysates were sonicated on ice using a cycle of 2 min (5 s of sonication followed by 2 s off), with an amplitude of 35%. Protein concentration was measured by BCA assay (Pierce (Thermo Fisher Scientific) #23227). Each sample underwent a click reaction using 150 µg of protein. The reagents for click chemistry were 50 mM TCEP, 50 mM CuSO_4_, and 5 mM biotin-alkyne (CCT-TA105). The total volume was 400 µL in PBS. The reaction was performed covered by aluminum foil, at room temperature for an hour, while rocking. Overnight, the samples were subjected to precipitation using acetone at a concentration of 80%. Afterward, the samples were washed with 95% cold ethanol and then dissolved again using 8M Gdn-HCl (200 µL). By performing a cycle of heat–freeze–vortex cycles, we ensured that most of the proteins were solubilized. All samples were verified for successful biotin conjugation by gel electrophoresis and probing with labeled avidin (Supplementary Figure S1).

### 2.5. Biotinylated Protein Capture

The clicked solubilized proteins were diluted to a concentration of 2M Gdn-HCl. The biotinylated proteins were trapped using Avidin Agarose (Pierce #20219) overnight with rotation. The capture efficiency was evaluated using a dot–blot technique. Then, 2 µL of both “pre” and “post” capture samples were added directly onto a nitrocellulose membrane and dried before immunoblotting (Supplementary Figure S2). The bead efficiency, measured by the fluorescence signal, uses the formula ((Pre-Post)/Pre) × 100). When the efficiency reached 70%, the beads were processed with an on-bead trypsin digestion as described [12].

### 2.6. Mass Spectrometry for AHA Labeling

The separation and the analysis of tryptic peptides were performed using a Vanquish Neo UHPLC system (Thermo Fisher Scientific) and an EASY-Spray capillary HPLC column (ES75150, Thermo Fisher Scientific) attached to an Orbitrap Exploris 480 mass spectrometer (Thermo Fisher Scientific). The mass spectrometer was conducted in data-dependent acquisition mode with a resolution of 60,000 and *m*/*z* range of 350–1700. Multiply charged ions were segmented via HCD dissociation with an NCE of 28 and spectra of their segments were filed in the orbitrap at a resolution of 15,000. Data was interpreted using Proteome Discoverer (Thermo Fisher Scientific, version 3.0) and the databases were examined using SEQUEST (Thermo Fisher Scientific) against the H. sapiens proteome. Search parameters involved a strict false discovery rate (FDR) of 0.01, a relaxed FDR of 0.05, a precursor mass tolerance of 10 ppm, and a fragment mass tolerance of 0.01 Da. Peptides were explored with carbamidomethyl cysteine as a static modification and oxidized methionine and deamidated glutamine and asparagine as dynamic modifications. Gene ontology was used on proteins with significant changes in abundance (fold change > 2 and *p* < 0.05; see statistics below) using https://metascape.org (access on 12 November 2024) against the *H. sapiens* proteome.

The functional summary for the proteins list was obtained using https://www.proteomaps.net/ (accessed on 14 November 2024) relative to the *H. sapiens* proteome.

The raw data of the analysis is available at ftp://massive-ftp.ucsd.edu/v09/MSV000097076/ (access on 4 November 2025) and the raw quantified protein lists are provided in Supplementary Table S1.

### 2.7. Label-Free LC-MS/MS

For the initial shotgun proteomic analysis, RPMI-8226 cells were cultured in 10 cm dishes until they reached a density of 500,000 cells/mL. Cells were treated with hydroxychloroquine (200 µM), Bortezomib (6.25 nM), or a combination of both. Cell lysates were resolved on a 10% SDS-PAGE gel and visualized by Coomassie staining. Each gel lane was excised into small slices, and proteins in each slice were subjected to in-gel tryptic digestion following established protocols [13]. The resulting peptide mixtures were vacuum-dried and reconstituted in solvent A (5% acetonitrile in 0.2% formic acid) for LC–MS/MS analysis.

Tryptic peptides were separated over a 60 min gradient using a Vanquish Neo UHPLC system (Thermo Fisher Scientific) with an EASY-Spray PepMap Neo column (75 µm × 150 mm, Thermo Fisher Scientific) coupled with an Orbitrap Exploris 480 mass spectrometer (Thermo Fisher Scientific). The mass spectrometer was operated in data-independent acquisition (DIA) mode. MS scans were acquired at a resolution of 60,000 over an *m*/*z* range of 350–1000, and DIA scans were performed at a resolution of 30,000 with a precursor mass range of 350–1000, a scan range of 145–1400, and isolation windows of 36.0 *m*/*z*. Ions were fragmented via HCD with a normalized collision energy (NCE) of 30. Data were processed and protein quantification was performed using Spectronaut (v18.5, Biognosys, Schlieren, Switzerland).

The raw data of the analysis is available at ftp://massive-ftp.ucsd.edu/v09/MSV000097478/ (accessed on 4 November 2025) and the raw quantified protein lists are provided in Supplementary Table S2.

### 2.8. Statistics and Data Analysis

For the label-free DIA analysis, the raw data was normalized to the total ion current and missing values were filled using the local minima for each sample as previously described [14]. The statistical comparison of proteins the three treatment and control groups was performed using a one-way Anova (Supplementary Table S5) and adjusted the *p*-values with a Benjamini–Hochberg *p*-value correction.

For AHA metabolic labeling proteomic analysis, protein lists were extracted to Microsoft Excel. Protein abundance was determined by looking at each protein’s extracted ions intensities. After background subtraction using the no-AHA control data, a global normalization was applied, and a global minimum was performed to zero-fill the missing values. Statistically significant differences were settled using linear regression at 5% significance for the degradation data. A maximal half-life of 25 h was set as this was four-fold of the time of the experimental data collection (Supplementary Table S3). For the protein synthesis data, an unpaired T-test was applied (Supplementary Table S4). For the synthesis data, a >2-fold change threshold was also applied to prioritize proteins with the largest effect sizes, as detailed below.

As a result of the challenges in multiple testing corrections in proteomic analysis, we have additionally employed the utilization of a stringent fold change criterion as a final filtering step. Specifically, we used a greater than >2-fold change as a cut-off to focus on proteins with the most robust effects. The appropriateness of this threshold was confirmed by analyzing the effect size distribution for the synthesis data (Supplementary Figure S3), which revealed that the >2-fold cut-off effectively excludes over 90% of the data points, isolating the non-linear portion of the distribution and minimizing statistical noise. This approach serves to minimize statistical errors by concentrating on large effect sizes, as previously described [15]. The same >2-fold change cut-off was utilized for the degradation rates. Although Supplementary Figure S4 demonstrates more proteins exceeding this cut-off, we maintained this consistency across both datasets to facilitate direct comparison, a decision further supported by our discussion of increased autophagy in the Results section.

### 2.9. Western Blot Analysis

Proteins were extracted using 1% SDS/PBS supplemented with a protease and phosphatase inhibitor cocktail. Protein concentration was determined using the BCA assay. Equal amounts of protein (10 µg) were resolved on a 12.5% SDS-PAGE gel and transferred onto a nitrocellulose membrane. The membrane was blocked with 2.5% fish skin gelatin in PBST for 1 h at room temperature and then incubated overnight at 4 °C with the primary antibody NEDD8 rabbit mAb (Cell-Signaling (Boston, MA, USA) #2754T) with dilution 1:2000. After washing, the membrane was incubated with an IR dye-conjugated anti-rabbit secondary antibody for 1 h at room temperature. Detection was performed using LI-COR imaging.

## 3. Results

### 3.1. Characterization of Bortezomib Treatment on Cell Viability

**Figure 1:**
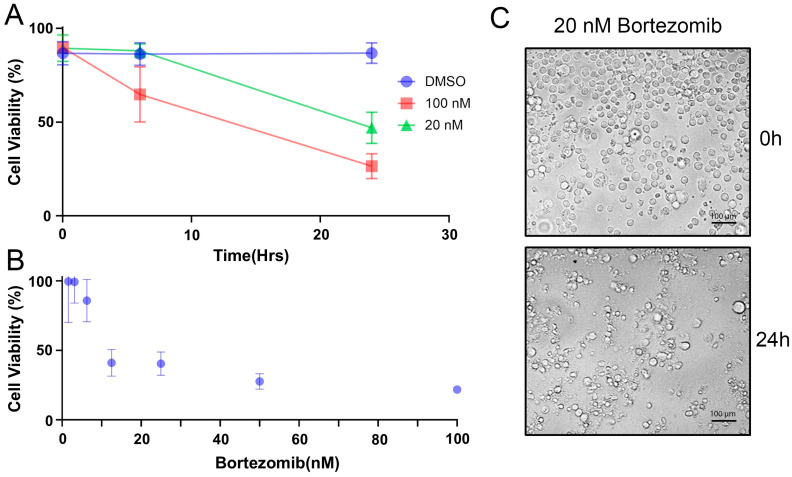
Cell viability following Bortezomib treatment. The cell viability of RPMI cells treated with varied doses of Bortezomib was evaluated using trypan blue staining (**A**) and the CCK-8 test (**B**), respectively. The data shows the mean ± standard deviation of three different experiments. (**C**) Visual confirmation by light microscopy of the cellular response of cells treated with 20 nM Bortezomib.

Prior to selecting Bortezomib treatment concentrations and time for proteomic analysis, the response of RPMI-8226 to Bortezomib treatment was assayed by cell viability (trypan blue exclusion) and metabolic activity (CCK-8 assay). Treatment of cells for 24 h with 0, 20, and 100 nM Bortezomib revealed that concentrations at 20 nM and higher resulted in >50% loss in cell viability, as quantified by trypan blue exclusion (Figure 1A). While we have not verified that this concentration of Bortezimib results in accumulation of polyubiquinated proteins as predicted upon effective proteasome inhibition, sensitivity to 20 nM Bortezimib with viability assays agrees with other published reports on RPMI-8226 cells [16,17]. Subsequently, a time course analysis of Bortezomib was investigated using a CCK-8 assay. As depicted in (Figure 1B), a significant reduction in the CCK-8 signal is similarly observed at 24 h treatment when compared to the data in (Figure 1A). At 8 h of treatment with Bortezomib, the 100 nM treatment exhibits some reduction in CCK-8 signal while no detectable change from the control is observed for 20 nM Bortezomib. As a final confirmation of treatment, the cells treated with 20 nM Bortezimib were imaged by light microscopy and obtained at 0 and 20 h of treatment (Figure 1C). As a result, it was decided to conduct the proteomic analysis at time points under 8 h at 20 nM Bortezomib to capture the earlier proteomic changes that eventually lead to cell death but excluded the confounding issues of containing significant fractions of dead cells.

### 3.2. Nascent Peptide Proteomics

**Figure 2:**
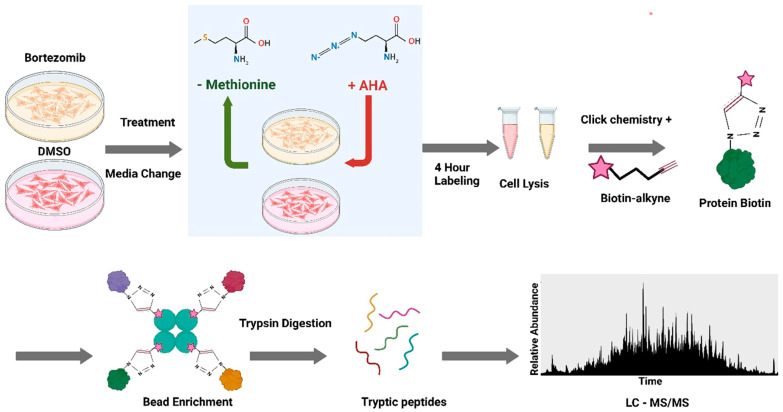
Workflow for examining Bortezomib-induced changes in protein synthesis. An AHA pulse–chase labeling method was used to investigate alterations in protein synthesis following Bortezomib treatment. After drug administration, cells were incubated in an AHA-containing medium for 4 h. The lysates underwent a biotin click chemistry reaction, and the biotinylated proteins were enriched using streptavidin beads prior to trypsin digestion. The resulting tryptic peptides were then analyzed by LC-MS/MS.

To characterize the proteins synthesized by RPMI-8226 upon Bortezomib treatment, we adopted the method where the methionine analog azidohomoalanine (AHA) is metabolically incorporated into the newly synthesized proteins as previously described [12]. As outlined in (Figure 2), this approach results in a unique chemical label being incorporated into nascent proteins that can be used for chemical derivatization and enrichment for proteomic analysis.

Upon the addition of DMSO (control) or Bortezomib (20 nM) to cells, 50 µM AHA was included to be incorporated into nascent proteins during the time of treatment. Additional controls with no AHA are included for background correction, where the background signal arises from both nonspecific binding and endogenously biotinylated proteins. After four hours of treatment, the cells were lysed, and the lysates were derivatized with biotin-alkyne. Derivatization with biotin was verified by probing lysates with labeled streptavidin after resolving by SDS-PAGE (Supplementary Figure S1). Biotinylated proteins were then isolated on streptavidin beads; validation of bead capture efficiency was determined by dot–blot analysis (Supplementary Figure S2) and the beads were then trypsinized for proteomic analysis as described in Materials and Methods.

**Figure 3:**
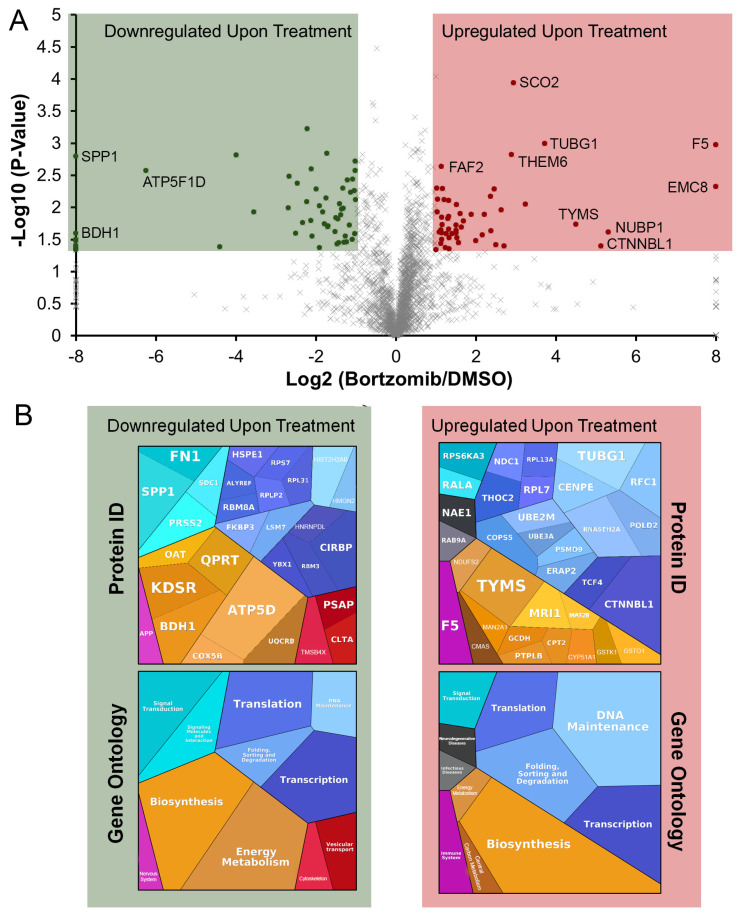
Proteome-wide changes to nascent protein synthesis in response to Bortezomib treatment. (**A**) Volcano plot depicting changes to protein synthesis in multiple myeloma cells upon Bortezomib treatment. The *x*-axis represents the fold change in protein abundance, and the *y*-axis represents the statistical significance of these changes as –log_10_ (*p*-value). Measurements were from quadruplicate samples. (**B**) Proteomap summary depictions of the upregulated and downregulated proteins by both protein ID and gene ontology function. Areas are proportional to the fold changes in protein amounts observed.

Proteomic analysis led to the identification of 2448 proteins (Supplementary Table S1). A statistical comparison of the proteins from the control and Bortezomib-treated cells is summarized in the Volcano plot depicted in (Figure 3A). Using cut-offs of *p*-values of <0.05 and a fold change of >2, we identified 58 proteins with increased protein synthesis and 53 with decreased protein synthesis during the 4 h 20 nM Bortezomib treatment. To summarize these up- and downregulated proteins, proteomaps were generated [18] using the fold change for area for the up- and downregulated proteins (Figure 3B).

### 3.3. Proteins Exhibiting Increased Synthesis upon Bortezomib Treatment

**Figure 4:**
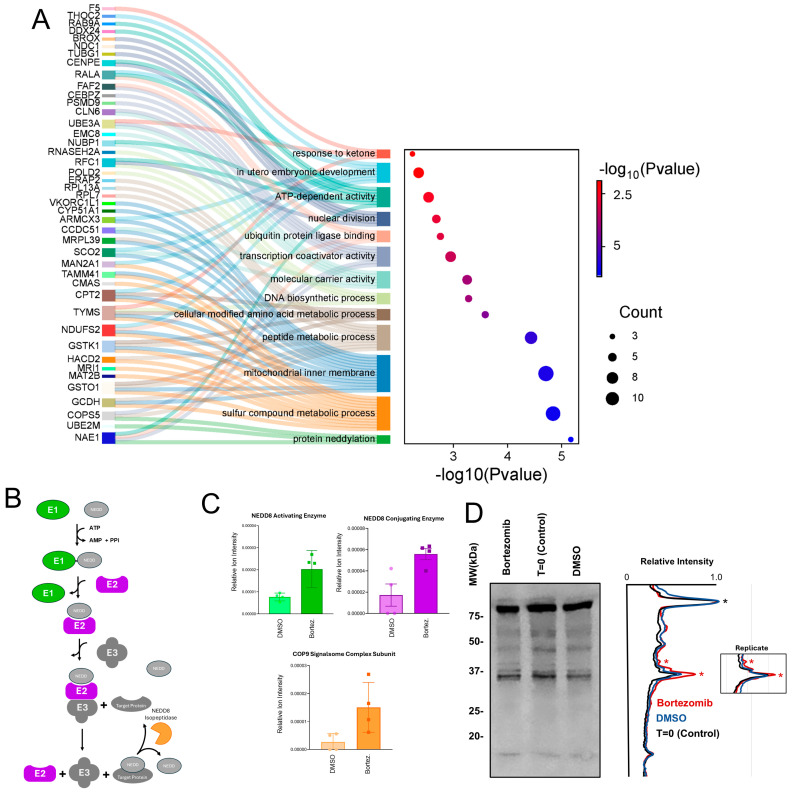
Gene ontology enrichment and protein neddylation. (**A**) Sankey and dot plots showing significant upregulation of gene ontology (GO) terms upon the Bortezomib treatment. The Sankey diagram (**left**) connects enriched GO terms to their associated genes, while the dot plot (**right**) represents statistical significance (−log_10_ (*p*-value)) by the horizontal coordinate and the color coding. The dot size represents the number of genes involved in each enriched term, highlighting the protein neddylation process as highly significant. (**B**) Schematic illustration of the major enzymes of the neddylation process; NEDD8-activating enzyme, NEDD8-conjugating enzyme Ubc12, and the COP9 signalosome complex subunit. (**C**) NEDD8-activating enzyme (E1), NEDD8-conjugating enzyme Ubc12 (E2), and the COP9 signalosome complex subunit (NEDD8 isopeptidase) were upregulated after 4 h of Bortezomib treatment, color-coded to correspond with panel B. (**D**) Immunoblot analysis showing the upregulation of some endogenous neddylation proteins upon the Bortezomib treatment in comparison with control. The right panel reveals the densitometry of the quantified blots, with an insert revealing the trace of and an additional replicate analysis. Profile peaks revealing alterations in neddylation are highlighted (*).

To characterize the biological processes associated with these 58 proteins during Bortezomib treatment, gene ontology (GO) enrichment analysis was conducted. The results of the GO enrichment analysis shown in (Figure 4A) reveal 13 statistically significant GO terms with protein neddylation exhibiting the lowest *p*-value.

The three proteins identified with GO annotations of protein neddylation that increase in abundance upon Bortezomib treatment are NEDD8-conjugating enzyme Ubc12, NEDD8-activating enzyme, and COP9 signalosome complex subunit. The function of these proteins in neddylation is summarized in (Figure 4B) and the quantification of their increased synthesis is depicted in (Figure 4C). With increased synthesis of proteins involved in both neddylation and de-neddylation, we hypothesized that global protein neddylation may be impacted by Bortezomib treatment. The increase in NEDD8-conjugating and -activating enzymes could lead to increased neddylation while conversely increased COP9 could potentially result in reduced protein neddylation.

To investigate whether Bortezomib treatment alters global protein neddylation, we analyzed protein lysates from cells treated with 20 nM Bortezomib for 4 h and compared them to DMSO-treated controls. We resolved the lysates via SDS-PAGE, transferred them to nitrocellulose membranes, and immunoblotted them with an anti-NEDD8 antibody. As shown in Figure 4D, while the most abundantly neddylated proteins remain unchanged, densitometric analysis reveals alterations in the neddylation of some other detectable proteins, suggesting that Bortezomib has a specific, rather than a broad, impact on this pathway.

### 3.4. Proteins Exhibiting Decreased Synthesis upon Bortezomib Treatment

**Figure 5:**
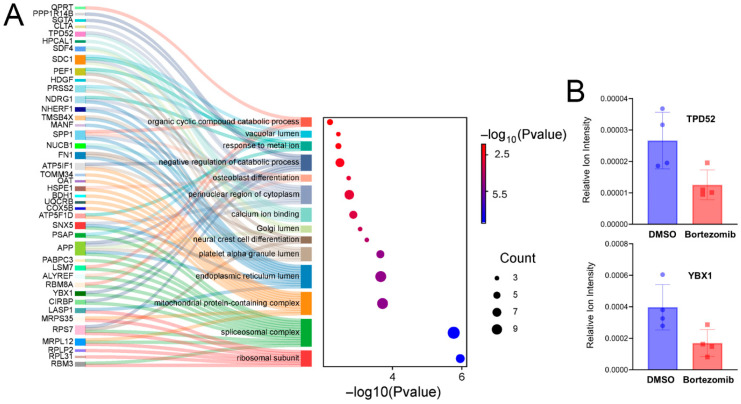
Gene ontology enrichment analysis of downregulated proteins upon Bortezomib treatment. (**A**) Sankey and dot plots show the enriched gene ontology pathways linked with proteins that are downregulated following Bortezomib treatment. The 14 most prominent clusters are displayed together with their corresponding augmented terms. Dot size is directly proportional to the quantity of genes associated with the ontology term. Dot color and their location on the *x*-axis is the −log_10_ (*p*-value) for the enrichment analysis. (**B**) Representative examples of downregulated proteins during the drug treatment include YBX1 and TPD52.

To characterize the biological processes associated with these 53 proteins during Bortezomib treatment, gene ontology (GO) enrichment analysis was conducted. The results of the GO enrichment analysis shown in (Figure 5A) reveal 14 statistically significant GO terms with those involved in aspects of gene expression (Spliceosomal complex and Ribosomal proteins) being the most statistically significant. The insert in (Figure 5B) reveals the quantified proteomic data for two example proteins, YBX1 and TPD52, which are discussed in the following sections.

### 3.5. Pulse–Chase Protein Degradation Proteomics

**Figure 6:**
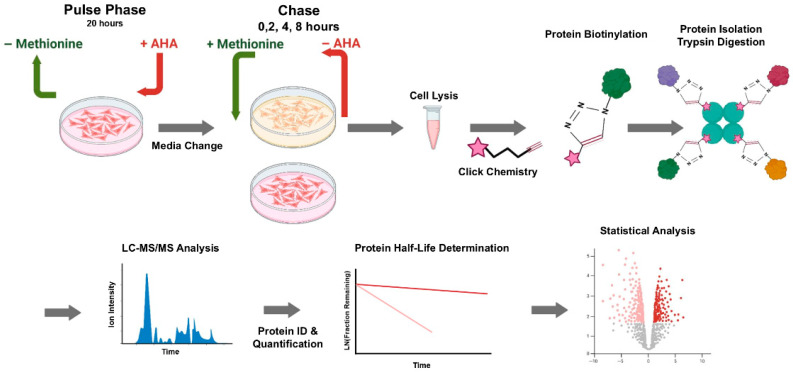
Workflow for AHA labeling to measure protein degradation. A schematic illustration of the AHA pulse–chase method used to study protein degradation, including the data transformation to determine and compare protein half-lives.

To investigate the impact of Bortezomib treatment on global protein degradation an altered AHA labeling method was utilized. As outlined in (Figure 6), metabolic “Pulse” labeling with AHA is performed prior to treatment. After labeling, AHA is removed and Bortezomib (20 nM) or DMSO (control) is added to the media as a “Chase”. AHA-labeled proteins are then quantified over time to monitor their disappearance.

RPMI-8226 cells were metabolically labeled overnight with 50 µM AHA upon which the media was changed to remove the AHA and the cells were then treated with Bortezomib (20 nM) or DMSO (control). Additional controls with no AHA are included for background correction. At time points performed in triplicates of 0, 2, 4, and 8 h, the cells were harvested and lysed. Lysates were derivatized with biotin-alkyne. Derivatization with biotin was verified by probing lysates with labeled streptavidin after resolving by SDS-PAGE (Supplementary Figure S1). Biotinylated proteins were then isolated on streptavidin beads; validation of bead capture efficiency was determined by dot–blot analysis (Supplementary Figure S2) and the beads were then trypsinized for proteomic analysis as described in Materials and Methods.

### 3.6. Determination of Protein Half-Life After AHA Pulse–Chase

**Figure 7:**
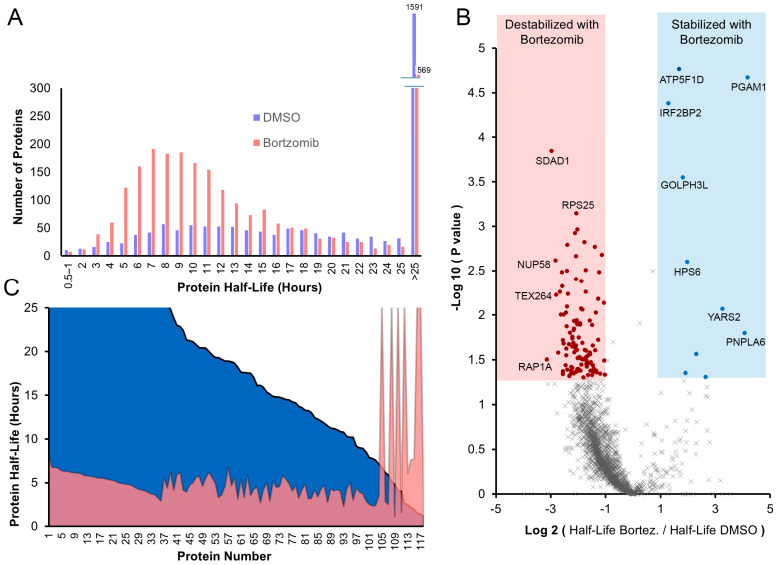
Impact of Bortezomib on protein degradation. (**A**) Binning diagram displaying the half-lives of proteins treated with DMSO and Bortezomib reveals the frequency of different protein half-lives observed. (**B**) Volcano plot comparing half-lives of proteins in treated versus untreated cells. The *x*-axis represents fold changes in protein half-lives, while the *y*-axis represents the statistical significance of this change as –log_10_ (*p*-value). (**C**) Plot depicting the distribution of protein half-lives of protein exhibiting significant differences between the DMSO (blue) and Bortezomib (pink) treatments.

The background subtracted protein abundance data collected at the initial 0 h time points are used to calculate the fraction of proteins remaining for the 2, 4, and 8 h time points. Three samples were measured for each time point. The natural logarithm of the fraction remaining was then computed to facilitate the calculation of protein half-lives. This analysis was performed on the 2448 proteins identified by LC-MS/MS analysis. As a result of the data being over an 8 h time course, a maximal limit to half-life determination of 25 h was set. The range of calculated protein half-lives for the treated and untreated cells is depicted in (Figure 7A). Of note is the revelation of an increase in abundance of proteins exhibiting shorter half-lives upon Bortezomib treatment.

Linear regression analysis was applied to the transformed data to identify statistically significant changes in protein stability between control and Bortezomib treatments. By applying a statistical cut-off of <0.05 and >2-fold change as depicted in (Figure 7B), 108 proteins were identified to be destabilized upon Bortezomib treatment and only 10 were stabilized. The 10 proteins identified to exhibit increased stability are listed in Table 1. A visual depiction of the fold changes of 118 proteins is summarized in (Figure 7C).

### 3.7. Diverse Protein Groups Degraded upon Bortezomib Treatment

**Figure 8:**
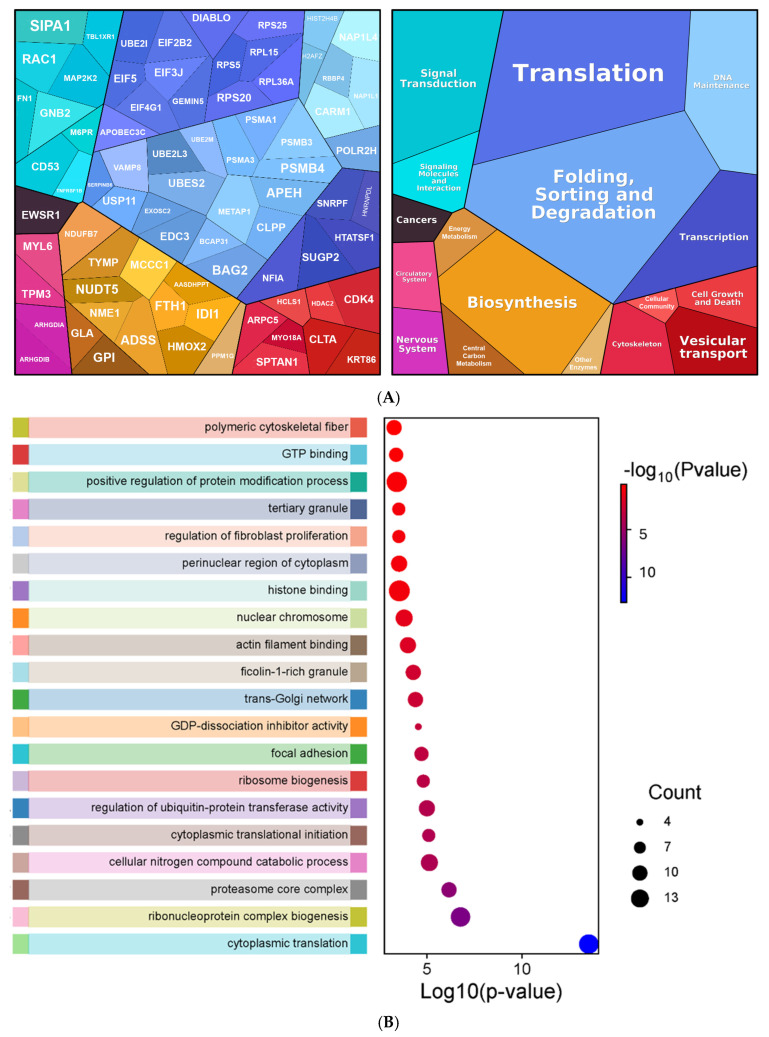
Destabilized proteins under Bortezomib treatment. (**A**) Proteomaps depicting the significantly destabilized proteins following Bortezomib treatment for protein ID and GO functions. The area of each polygon is proportional to the fold change observed. (**B**) Sankey and dot plot summarizing the enriched gene ontology pathways. The top 20 clusters are displayed together with their representative enriched terms. Dot size corresponds to the number of genes associated with the ontology term, while the *x*-axis and color scale represent the −log_10_ (*p*-value).

Proteomaps of the 108 Bortezomib-destabilized proteins (Figure 8A) reveal diverse functions of the destabilized proteins. Subsequent GO enrichment analysis of these proteins (Figure 8B) mirrors these broad categories including cytoplasmic translation, ribonucleoprotein complex biogenesis, and proteasome core complex.

A closer examination of the data suggests that the extent of increased protein degradation induced by Bortezomib treatment may be significantly greater than listed with our cut-offs, potentially due to Type II statistical errors. In the volcano plot (Figure 7B) and Supplementary Figure S4, a large fraction of the observed proteins appears to have decreased stability upon Bortezomib treatment (observed as the apparent asymmetry in the plot) but did not pass the statistical tests. This may be due to data noise introduced by the bead-based enrichment method, which tends to produce higher background signals compared to whole-proteome analysis. This widely appreciated issue of bead enrichment proteomics has led to databases such as the “CRAPome” [19,20].

### 3.8. Investigating the Role of Autophagy in Protein Degradation

With the apparent extensive increase in protein degradation upon Bortezomib treatment, we hypothesized that another mechanism of protein degradation must be actively leading to increased protein degradation. We hypothesized that this may be autophagy as proteasome inhibition has been documented to enhance this protein degradation pathway [21,22,23].

**Figure 9:**
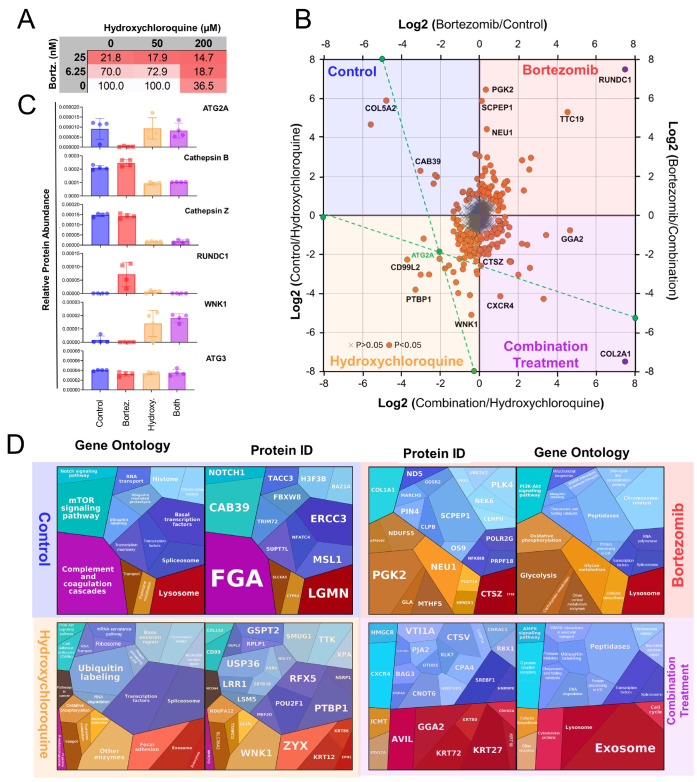
Label-free proteomics analysis. (**A**) CCK8 cell viability assay quantifying the impact of different Bortezomib and hydroxychloroquine concentrations on RPMI cells. Based on the results, concentrations of 6.25 nM for Bortezomib and 200 μM for hydroxychloroquine were chosen for the following experiments. (**B**) A four-way plot shows the fold changes for four conditions: Bortezomib, hydroxychloroquine, control, and combination treatment. Orange dots represent proteins that exhibit a *p*-value of <0.05 from a one-way Anova and are 1 unit away from the origin of the graph. The green dot highlights ATG2 as an example for triangulation; green dashed lines demonstrate the intersection of the data from the four axes, representing its relative change across all conditions. The purple dots represent proteins exhibiting larger differences that are beyond the scale of the graph and are artificially positioned to facilitate their identity and quadrant location. Each measurement was made with quadruplicate samples. (**C**) The panel shows the change in the relative abundance of selected proteins under the four treatment conditions. (**D**) Proteomap summaries of protein IDs and GO function for all four treatment conditions. The area of the polygon correlated to the fold change (distance from the origin) for each protein.

Initially, we investigated the potential synergistic effects of Bortezomib treatment with hydroxychloroquine, an inhibitor of autophagy that inhibits the acidification of lysosomes [24]. RPMI-8226 cells were investigated by the CCK-8 assay after 24 h of treatment with individual and combinations of Bortezomib (0, 6.25, and 25 nM) and hydroxychloroquine (0, 50, and 200 μM). The results of the assay from this treatment matrix are shown in (Figure 9A), from which drug concentrations of 6.25 nM Bortezomib and 200 μM hydroxychloroquine were selected for subsequent experiments.

### 3.9. Label-Free Proteomic Analysis

**Figure 10:**
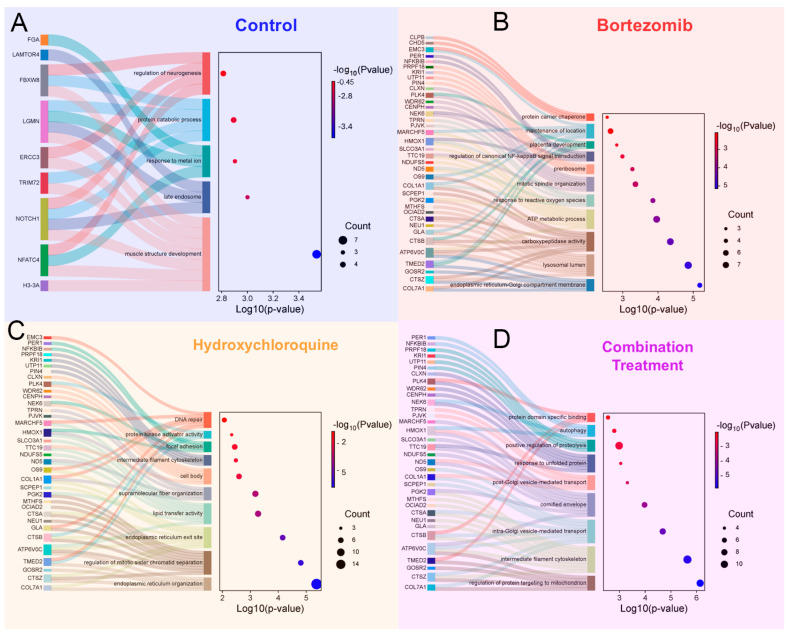
Gene ontology enrichment for label-free proteomics. (**A**) Sankey dot plot depicting gene ontology enrichment for the control treatment. The left panel represents enriched terms with their related genes, while the right panel shows −log_10_ (*p*-value) on the *x*-axis and color coding. The dot size represents the number of genes associated with each term. (**B**) Same as in panel A, applied to Bortezomib-treated group. (**C**) Hydroxychloroquine. (**D**) Combination treatment.

Quadruplicate samples of RPMI-8226 cells were subjected to treatment with DMSO (control), 6.25 nM Bortezomib, 200 μM hydroxychloroquine, or both drugs combined for 8 h. Cells were then lysed and prepared for DIA proteomic analysis as described in the experimental procedures. The resulting proteomic data identified 4125 proteins, and the relative abundance of each protein quantified is summarized in the four-way plot in (Figure 9B). Using the protein ATG2A as an example, the green lines depict how the coordinates of a protein are determined for the four-way comparisons. This visual representation highlights which proteins are most abundant in each treatment group or control. The quantified data for six proteins with reported links to autophagy are depicted in (Figure 9C), which includes ATG2A, cathepsin B, cathepsin Z, RUNDC1, WNK1, and ATG3. The triangulation of the statistically significant proteins in the four-way plot results in individual proteins being located in one of the four quadrants of the graph. These proteins and their GO functions for the proteins from each graph quadrant are depicted in the four proteomaps in (Figure 9D). Subsequent GO enrichment analyses of the statistically significant proteins in each quadrant are depicted in (Figure 10).

## 4. Discussion

Prior to applying the AHA methodology to investigate protein synthesis or protein degradation, the characterization of RPMI-8226 cell’s response to Bortezomib treatment was investigated with the CCK-8 assay and further verified by the trypan blue viability assay (Figure 1). Our results of Bortezomib’s dose-dependent induction of apoptosis are in accordance with previous studies [25]. Our data revealed a 50% reduction in cell viability of RPMI-8226 cells after 24 h of treatment using a 20 nM drug concentration, but no statistical change to viability was observed at 8 h of treatment (Figure 1A). As a result, we chose 8 h as a maximal time point for investigations with AHA as we wanted to investigate cellular processes occurring prior to significant cell death.

### 4.1. Impact of Nascent Protein Synthesis

As outlined in (Figure 2), we used AHA metabolic labeling during Bortezomib treatment to metabolically label only the proteins synthesized during drug exposure. This was a method previously reported for the labeling of nascent proteins [12]. As summarized in (Figure 3A), of the 2448 proteins identified only 111 (~4.5% of identified proteins) were determined to have altered protein synthesis upon Bortezomib treatment using a criterion of a *p*-value of <0.05 and an average change in abundance of >2-fold.

The initial summary of the proteins exhibiting increased and decreased synthesis upon Bortezomib treatment are summarized in the proteomaps in (Figure 3B). Notably, the proteomaps are not a complete summary as this software platform was unable to map functions for some proteins in the decreased synthesis group and in the upregulated group. Nonetheless, proteomaps reveal there is generally a lack of unifying process changing in concert, where proteins involved in translation, protein folding, and signal transduction are observed in both increased and decreased protein synthesis groups.

### 4.2. Increased Synthesis of Proteins

A deeper analysis of the proteins with increased synthesis in response to Bortezomib treatment by a GO enrichment analysis (Figure 4A) revealed 13 enriched GO terms. As Bortezomib inhibits the ubiquitin proteasome system, the proteins associated with the ubiquitin protein ligase binding GO term were explored for reported associations to biochemical pathways relevant to multiple myeloma (MM). FAF2 (also known as UBXD8) was initially identified as a protein involved in ERAD (endoplasmic reticulum-associated protein degradation) [26]. RALA, a member of the RAS superfamily of small GTPases, has been reported to be upregulated in MM and has been suggested as a potential target for the disease [27]. NDUFS2, a subunit of mitochondrial complex I, has no reported association with MM we are aware of at this time but has recently been reported to play a role in pancreatic cancer [28]. NAE1 is the NEDD8-activating enzyme and is discussed below.

#### Protein Neddylation

The GO term with the lowest *p*-value from the analysis was protein neddylation (Figure 4A) which included the proteins NAE1, UBE2M, and COPS5. As depicted in (Figure 4B), these three proteins include the NEDD8-activating and -conjugating enzymes in addition to a subunit of the COP9 signalosome complex involved in de-neddylation. At first glance, the upregulation of proteins involved in both neddylation and de-neddylation appears contradictory, but previous reports mirror this apparently contrarian observation as COPS5 has been identified to have a critical role in MM [29]. In addition, MLN4924, a potent and highly selective small molecular inhibitor of NAE, had a synergistic effect with Bortezomib on different models of multiple myeloma [30]. While our data is suggestive of changes in protein neddylation, the data did not lead to a direct prediction to how it may be altered.

As the proteomic data was suggestive of potential changes in protein neddylation, we investigated whether changes in neddylation occurred. Immunoblotting for global protein neddylation with a NEDD8-specific antibody in (Figure 4D) did not reveal obvious changes to the most abundant neddylated protein species; some changes were observed for minor protein species around the 37 kDa MW size. Our observations of altered neddylation upon Bortezomib treatment are in agreement with previous detailed mechanistic investigations on neddylation with another proteasome inhibitor, MG132, and other proteotoxic stressors [31]. The identification of the neddylated proteins in our study was not pursued as this was beyond the scope of our study, but we hypothesize that neddylation-targeted proteomic analysis upon Bortezomib would reveal significant changes similar to that reported in other studies [31].

These findings have important therapeutic implications. The fact that Bortezomib treatment appears to elevate neddylation capacity suggests a potential adaptive mechanism by cancer cells to bypass proteasome inhibition. This is consistent with prior studies in multiple myeloma models that have shown that co-targeting neddylation with an inhibitor like MLN4924 can synergize with Bortezomib to enhance therapeutic efficacy [32]. Our data supports the hypothesis that Bortezomib may drive a cellular response to elevate neddylation, thus providing a strong rationale for exploring a combined treatment strategy that simultaneously targets both the proteasome and the neddylation pathway.

### 4.3. Decreased Protein Synthesis upon Bortezomib Treatment

GO enrichment analysis of the 53 proteins with reduced synthesis upon Bortezomib treatment (Figure 5A) revealed that the most statistically enriched terms suggest reduced synthesis of proteins involved in gene expression (Ribosomal subunit and splicing complexes) in addition to organellar proteins and some catabolic processes. Of these proteins, some have been previously identified to be relevant to MM. Tumor protein D52 (TPD52) has long been reported to often be upregulated in MM [33], and our study reveals a ~2-fold decrease in TPD52 abundance (Figure 5B) which may broadly impact cellular metabolism via its reported role in regulating AMPK [34] or impacting cellular secretion [35]. Y-Box binding protein (YBX1) has also a long history with MM and has been associated with disease progression and drug resistance [36]. The mechanism for the role of YBX1 in MM may be activated in part through its signaling axis with MYC [37].

### 4.4. Protein Degradation

Our objective was to investigate the impact of Bortezomib treatment on global protein stability in the RPMI-8226 MM model for the first time using the AHA pulse–chase method as outlined in (Figure 6). Prior to the study, we had a simple hypothesis that Bortezomib treatment would result in the stabilization of many proteins with short half-lives. The data summarized in (Figure 7) revealed essentially the opposite of what we predicted, where Bortezomib treatment resulted in the destabilization of many proteins (108) while a small number (10) were stabilized.

As will be further elaborated below, the majority of proteins were not determined to have altered stability upon Bortezomib treatment, but this may in part be a result of data variability obtained with the current iteration of the AHA-labeling method which requires significantly more handling steps when compared to whole-proteome analysis and challenges with bead-based proteomic methods as mentioned previously.

### 4.5. Stabilized Proteins upon Bortezomib Treatment

The ten proteins stabilized by Bortezomib treatment are listed in Table 1, which are too few for GO enrichment analysis. Nonetheless, from this limited list of proteins, a number of them have been implicated in autophagy and endoplasmic reticulum (ER) stress, which includes IST1 [38], IGF2BP2 [39], AP3D1 [40], HSP6 [41], and QRICH1 [42]. Taken together, the data is suggestive of possible impacts to autophagy and ER stress in response to Bortezomib treatment.

### 4.6. Destabilized Proteins upon Bortezomib Treatment

Treatment with Bortezomib led to the identification of 108 proteins where their half-lives decreased by >2-fold and a *p*-value of <0.05 when statistically comparing the slopes for rate determination. As can be seen in the volcano plot in (Figure 7B), it appears that the bulk of the proteome also seems to have been destabilized upon Bortezomib treatment but did not pass the statistical cut-offs. This shift in the bulk of the data has been evaluated to ensure it was not a result of data normalization as similar results are obtained using alternative calculations As a result of data variability, we hypothesize there are a significant number of Type II statistical errors (false negatives) in the analysis. As a result, the data presented on the range of protein half-lives in (Figure 7C) of all proteins presents an appropriate representation of the magnitude of the proteins being destabilized upon Bortezomib treatment.

The 108 proteins that met statistical cut-offs were analyzed for function and are portrayed in (Figure 8). As the GO function and GO enrichment analysis reveals, the cellular processes impacted are very diverse, including those that may be expected with Bortezomib treatment such as protein synthesis, proteasome function, and other processes involved in normal cell growth.

Overall, we hypothesized the apparent global protein degradation induced by Bortezomib treatment to be a result of the activation of autophagy. The linkage of autophagy to the ubiquitin proteasome system has been well-documented [20,43], and it has been previously discussed how proteotoxic stress from proteosome inhibition can lead to autophagy.

### 4.7. Investigating Autophagy and Proteasome Inhibition

To follow up on the interplay of autophagy and the ubiquitin proteasome system, we investigated hydroxychloroquine in combination with Bortezomib. As hydroxychloroquine prevents acidification of lysosomes, it can inhibit protein degradation by autophagy [44]. Our initial investigation was to determine which concentrations of Bortezomib and hydroxychloroquine would result in an additive response upon an overnight treatment (Figure 9A), as has been reported previously [45,46]. This resulted in the determination of 6.25 nM Bortezomib and 200 µM hydroxychloroquine. While our drug dilutions were limited, we were able to estimate a Bliss synergy score of ~2.3 which indicates the treatments are essentially additive in their cellular effect. Like the AHA metabolic labeling experiments, follow-up investigations on drug treatments were performed after a shorter treatment time (8 h) to capture protein changes prior to significant cell death.

Label-free proteomic analysis of the three drug treatments (Bortezomib, hydroxychloroquine, and combined) and control for the 4125 quantified proteins is depicted in the four-way plot (Figure 9B). This plot was designed to enable a four-way comparison where each protein is triangulated from the four different ratios between groups as depicted. The plot facilitates the visualization of whether proteins are observed in higher amounts as a result of specific treatments. As this experiment was initiated from our line of inquiry into autophagy, the data from the six proteins relevant to autophagy are shown in (Figure 9C). From these individual proteins, we observe five patterns of response: lower upon Bortezomib treatment, lower in hydroxychloroquine (or combined) treatment, higher upon Bortezomib treatment, higher in hydroxychloroquine (or combined) treatment, or unchanged in response to treatments. The lipid transfer protein ATG2A with reported roles in autophagy [47,48] is observed at reduced amounts upon Bortezomib treatment. The lysosomal cathepsin proteases are lower in hydroxychloroquine or combined treatment. The RUNDC1, a reported inhibitor of autolysosome formation [49], is upregulated upon Bortezomib treatment. The WNK lysine-deficient protein kinase 1 (WNK1), a reported inhibitor of autophagy [50,51], is higher in abundance upon hydroxychloroquine (or combined) treatment, while the E2-conjugating enzyme ATG3 for the covalent conjugation of LC3 phosphatidylethanolamine lipids during autophagy [52] remains unchanged upon any treatment.

While changes in some of these autophagy-related proteins suggest Bortezomib treatment impacts autophagy, our observed results are somewhat confounding. The decreased abundance of ATG2A upon Bortezomib treatment is perplexing given its role in autophagosome formation [53]. Similarly, the increase in RUNDC1 upon Bortezomib treatment is counter to its recently reported role in inhibiting autolysosome formation [54]. While our observations seem to counter some aspects of canonical autophagy, prior proteomic investigations have revealed a variant mechanism for lysosomal protein delivery of proteins independent of key autophagy factors [55]. Taken together, while our data supports broad-based protein degradation upon Bortezomib treatment, the mechanism on how this occurs may be a variant of autophagy which may eventually be elucidated with future investigations.

Cross referencing the selected proteins in (Figure 9C) with the AHA-labeling experiments highlights the limitation of the AHA method with respect to proteome coverage. The two proteins which exhibit changes between the control and Bortezomib treatments (ATG2A and RUNDC1) were both not detected in the AHA dataset. Cathepsin B, cathepsin Z, and ATG3 were all detected in the AHA datasets (Supplementary Table S1) but did not exhibit statistically significant changes between the two treatments as was observed for the label-free proteomic analysis.

Taken together, while our data supports broad-based protein degradation upon Bortezomib treatment and reveals potential impacts on autophagy, we cannot definitively conclude that other protein degradation mechanisms are not also involved. The precise mechanism by which this occurs may be a variant of autophagy or another uncharacterized process, which warrants further investigation.

### 4.8. Functional Outcomes of Drug Treatments

GO functional outcomes of the proteins altered upon the three different treatments also reveal aspects of organelle maintenance and trafficking. For the four quadrants of the four-way plot (Figure 9B), the GO enrichment of the quadrant representing Bortezomib treatment (Figure 10B) reveals ER-Golgi compartment membrane and lysosomal lumen proteins as the most significant GO terms. The quadrant for proteins enriched during hydroxychloroquine treatment include GO-enriched terms of ER organization, ER exit site, and lipid transfer activity as enriched terms (Figure 10C). Hydroxychloroquine treatment also revealed significant enrichment for proteins involved in DNA repair and mitotic sister chromatid separation. Lastly, the co-treatment with both drugs revealed GO enrichment of mitochondria-targeting, cytoskeleton, Golgi transport in addition to the response to unfolded proteins and autophagy (Figure 10D).

### 4.9. Future Considerations and Caveats

Although the evolving AHA metabolic labeling method enables novel investigations into diverse biological processes [56,57,58], it is essential to consider its inherent limitations. The substitution of methionine with the AHA analog during translation can subtly but significantly impact protein structure and function. As has been previously documented, these effects can include alterations to the overall rates of protein synthesis [59] and the induction of specific stress response pathways [60].

## 5. Conclusions

Here, we report for the first time the parallel use of proteomics and metabolic labeling of AHA to investigate either changes in protein synthesis or degradation in response to Bortezomib treatment. While the current methodology does not match the proteome coverage as traditional label-free proteomic analysis, the method provided insights that were not obtained from label-free analysis. For protein synthesis we observed Bortezomib treatment resulted in increased synthesis of enzymes central to protein neddylation. In contrast, for protein degradation investigations we observed destabilization of a broad cohort of proteins upon Bortezomib treatment, which led our lines of investigation to focus on a potential role for autophagy. Our subsequent investigations then focused on the proteome-wide changes in response to Bortezomib and hydroxychloroquine treatments. This label-free proteomic analysis did reveal broad impacts to the proteome as a result of treatments but not to the extent of the AHA-utilizing approaches despite the nearly two-fold increased proteome coverage (4125 proteins versus 2448) for the label-free analysis. Overall, it reveals that alternative proteomic methods complement traditional approaches when investigating complex processes such as proteasome inhibition. Future development of the technique may also provide future opportunities to investigate changes in proteoforms resulting from cellular responses, as the technique enables the isolation of newly synthesized proteins.

This study provides a foundational look into the proteomic response of multiple myeloma to Bortezomib. While our findings suggest novel therapeutic vulnerabilities, further investigations using a broader range of multiple myeloma cell lines and in vivo models are essential to validate these observations and to explore their full clinical potential.

## Figures and Tables

**Table 1:** Proteins stabilized upon treatment with Bortezomib.

Accession	Protein	Description	DMSO ½-Life (h)	Bortezomib ½-Life (h)
Q7Z5L9	IRF2BP2	Interferon regulatory factor 2-binding protein	2.5	6.1
P30049	ATP5F1D	ATP synthase subunit delta	1.1	3.6
Q2TAL8	QRICH1	Transcriptional regulator QRICH1	2.2	7.6
P53990	IST1	IST1 homolog	6.8	25.6
O14617	AP3D1	AP-3 complex subunit delta-1	1.9	7.7
Q9Y2Z4	YARS2	Tyrosine–tRNA ligase, mitochondrial	5.2	25.6
Q86YV9	HPS6	BLOC-2 complex member HPS6	4.1	25.6
Q9H4A5	GOLPH3L	Golgi phosphoprotein 3-like	2.7	25.6
Q8IY17	PNPLA6	Patatin-like phospholipase domain-containing protein 6	1.5	25.6
P18669	PGAM1	Phosphoglycerate mutase 1	1.4	25.6

## Data Availability

The raw data of the analysis is available at ftp://massive-ftp.ucsd.edu/v09/MSV000097478/ (accessed on 4 November 2025).

## Supplementary Materials

The following supporting information can be downloaded at https://www.mdpi.com/article/10.3390/proteomes13040063/s1. Figure S1: Validation of click reaction and protein loading by immunoblotting; Figure S2: Assessment of biotinylated protein capture efficiency via dot–blot; Figure S3: Analysis of Protein Synthesis Effect Size Distribution upon Bortezomib Treatment; Figure S4: Analysis of Protein Degradation Effect Size Distribution upon Bortezomib Treatment; Table S1: Raw protein list of proteins identified in AHA-labeling proteomics; Table S2: Raw protein list of proteins identified in label-free proteomic analysis; Table S3: The list of proteins with significantly altered protein degradation; Table S4: The list of proteins with significantly altered protein synthesis; Table S5: Four-way comparison of label-free proteomics; File S1: Uncropped gel images, dot blots, statistical effect size analysis for protein synthesis and protein degradation data.
