# Supplementary material for: Azidohomoalanine (AHA) Metabolic Labeling Reveals Unique Proteomic Insights into Protein Synthesis and Degradation in Response to Bortezomib Treatment
Source: Proteomes. 2025 Nov 25;13(4):63. doi: 10.3390/proteomes13040063 (PMC12737224; doi:10.3390/proteomes13040063)

Supplemental figure 1 :May 9/ 2024

Streptavidin blots for Multiple myeloma samples

Streptavidin blot,6.25 ug of protein each

No AHA 0 h 2 h/DMSO 2 h/Bortz 4 h/DMSO 4 h/Bortz 8 h/DMSO 8 h/bortz 4 h/B-R3

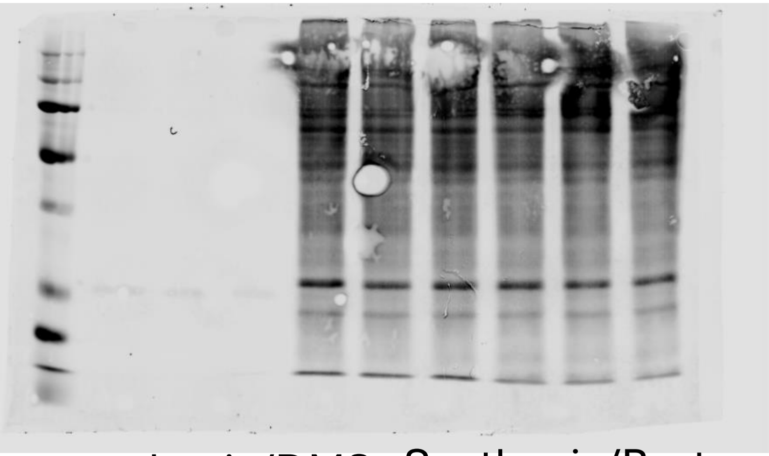

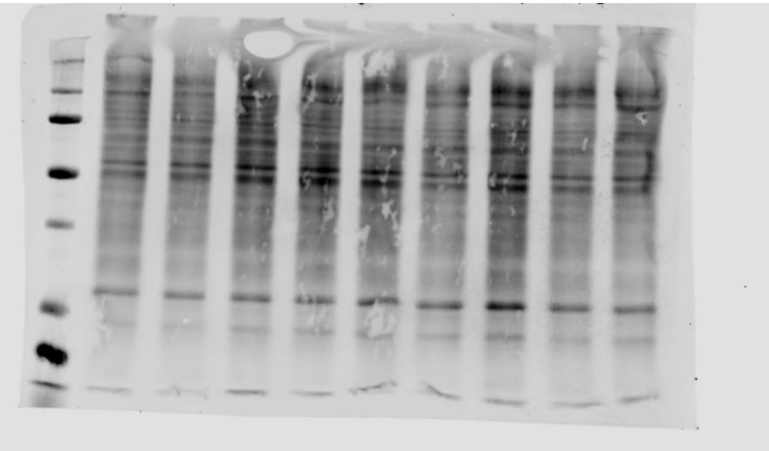

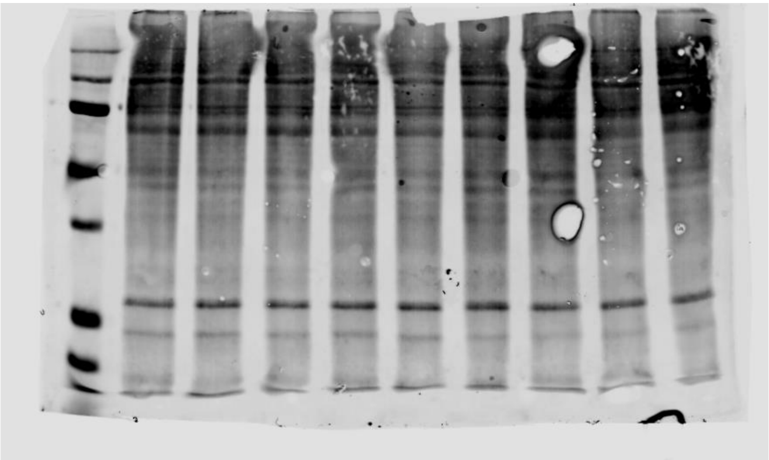

synthesis/DMSO Synthesis/Bortz

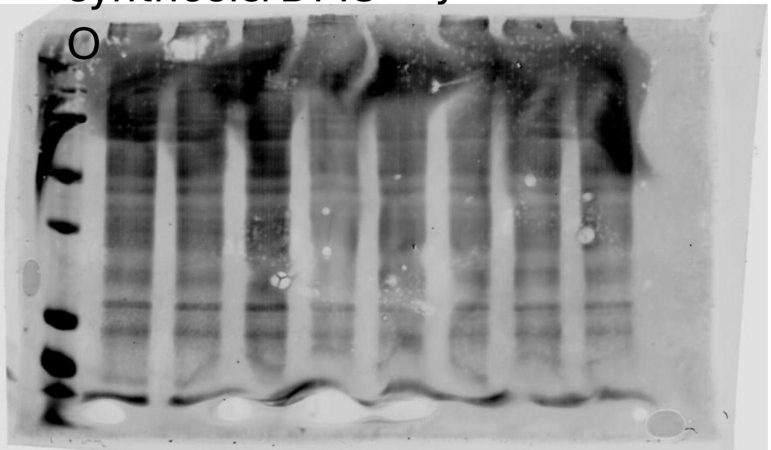

Supplemental figure 1 May 9/ 2024

Comassie blue stained gels for Multiple myeloma samples

Stained gels ,3 ug of protein each

No AHA 0 h 2 h/DMSO

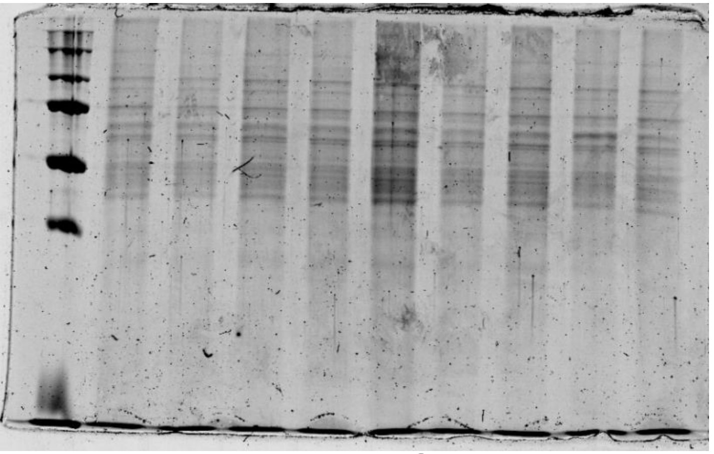

2 h/Bortz 4 h/DMSO 4 h/Bortz

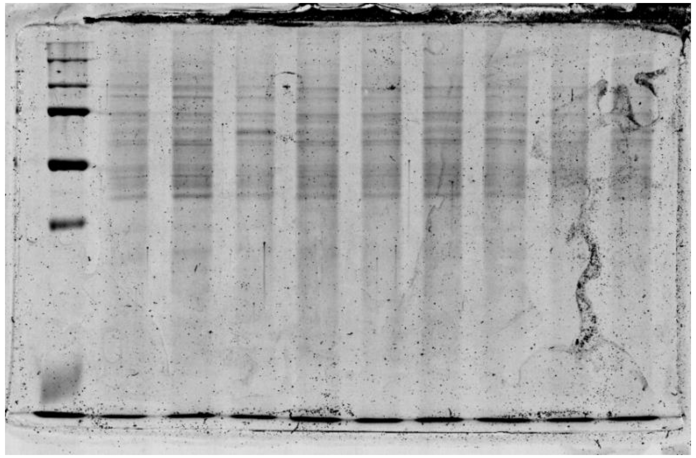

8 h/DMSO 8 h/bortz 4 h/B-R3

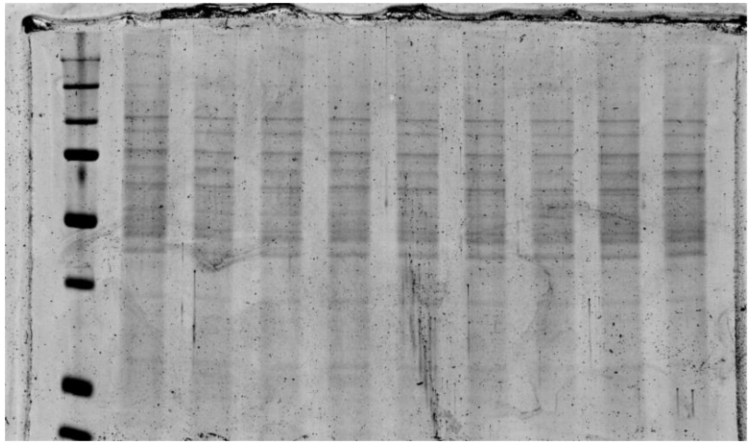

synthesis/DMS Synthesis/Bortz

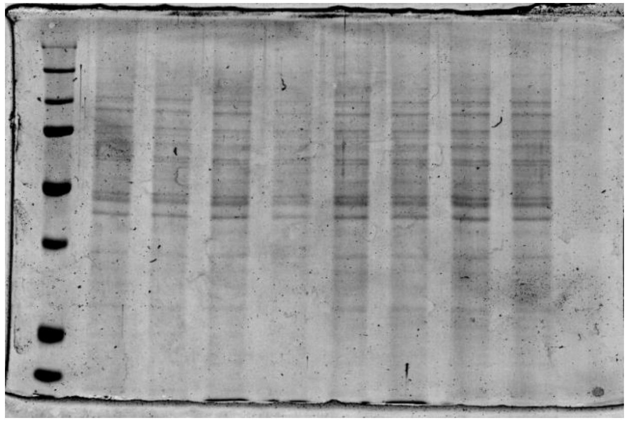

Supplemental figure 2

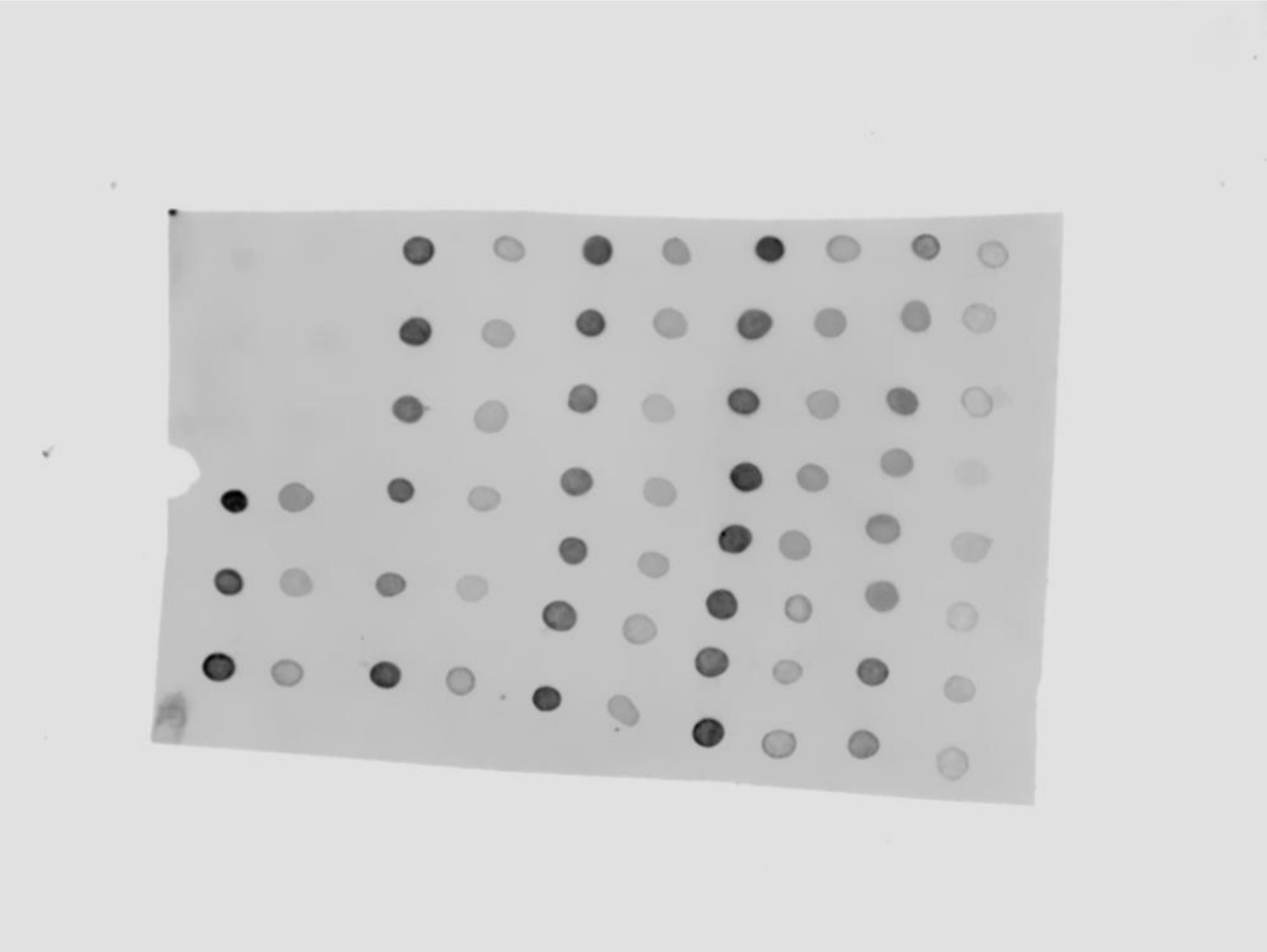

figure 4-D

Nedd8 blot

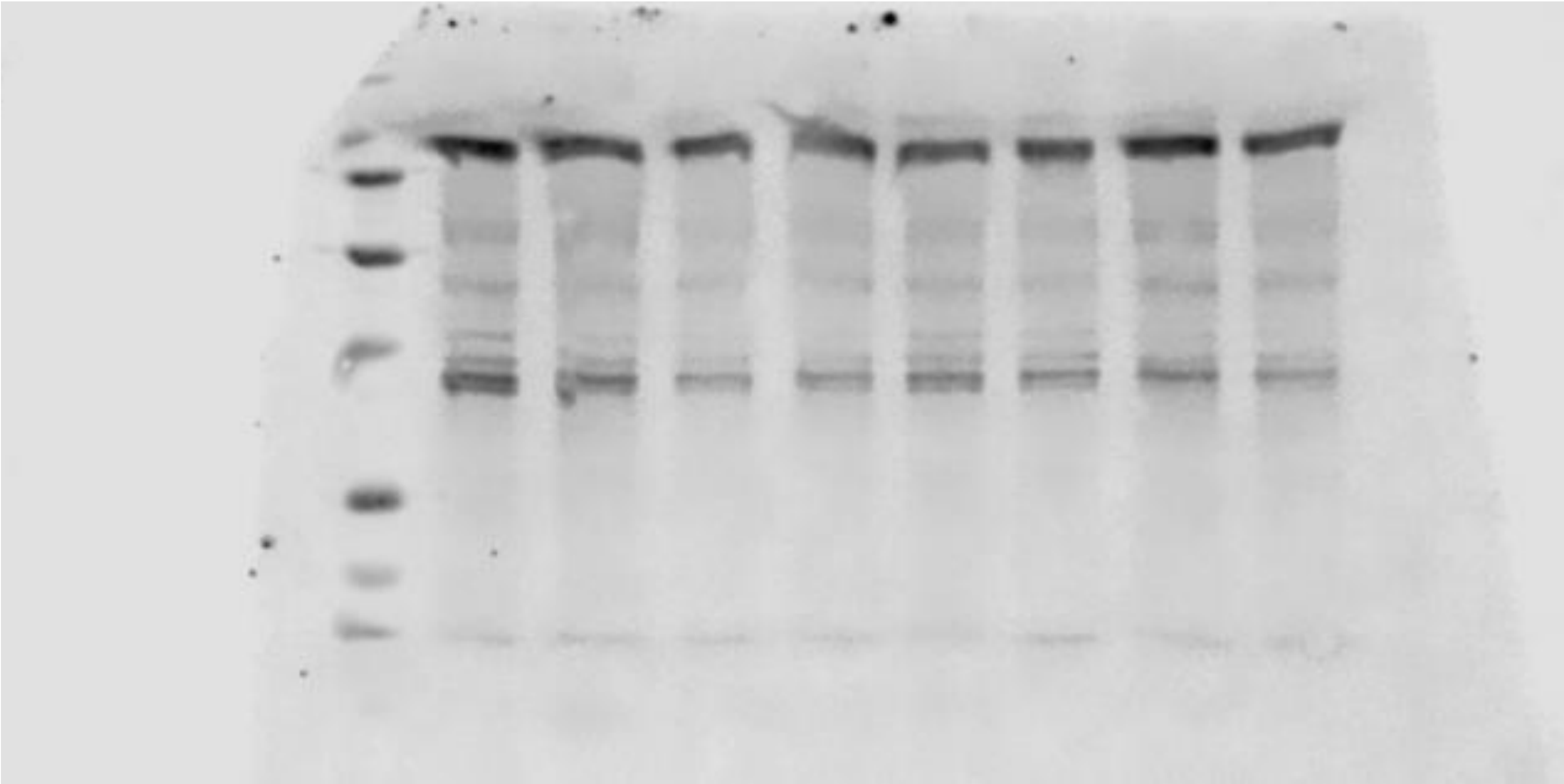

Supplement: Supplementary file 1 [file proteomes-13-00063-s001.zip › File S1. raw data immuno blots.pdf]
