# Supplementary material for: Azidohomoalanine (AHA) Metabolic Labeling Reveals Unique Proteomic Insights into Protein Synthesis and Degradation in Response to Bortezomib Treatment
Source: Proteomes. 2025 Nov 25;13(4):63. doi: 10.3390/proteomes13040063 (PMC12737224; doi:10.3390/proteomes13040063)
Supplement: Supplementary file 1 [file proteomes-13-00063-s001.zip › Supplementary materials figures.pdf]

Supplemental Figures.

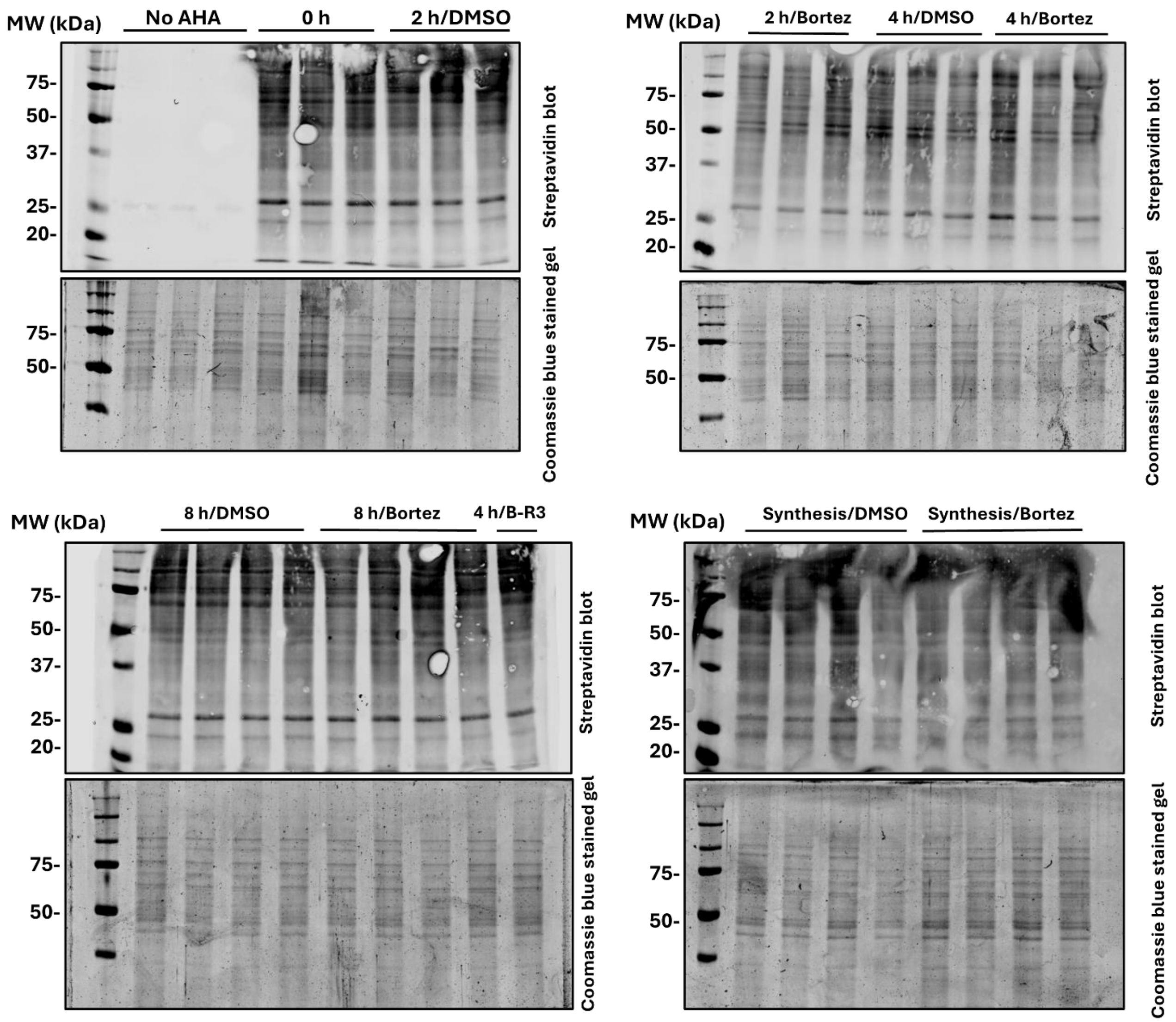

Figure S1: Validation of Click Reaction and Protein Loading by Immunoblotting. Immunoblots for all samples with their replicates were performed using 6.25 ug of protein per sample to verify the effectiveness of the click reaction. Following wet transfer, membranes were blocked for one hour with 2.5% fish skin gelatin, then incubated for one hour with streptavidin to verify the click reaction. All Coomassie blue-stained gels are attached to their streptavidin blots to validate that we uploaded the same amount of proteins for each lane.

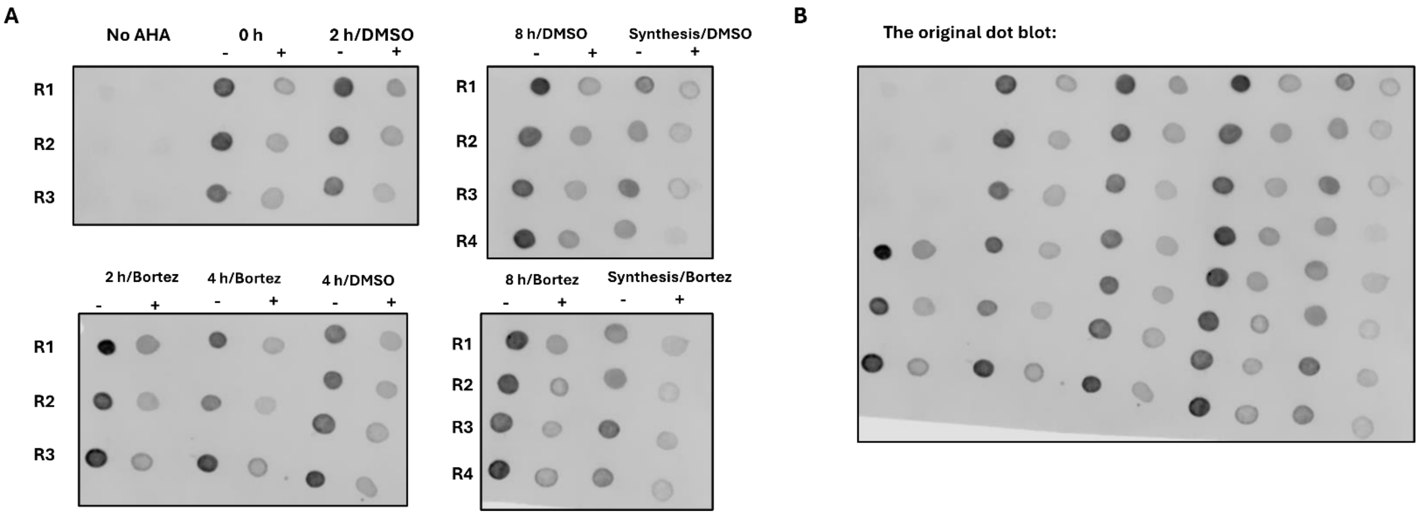

Figure S2: Assessment of Biotinylated Protein Capture Efficiency via Dot Blot. To evaluate streptavidin bead capture of biotin-labeled proteins. 2 ul of both pre-capture and post-capture samples were directly uploaded onto a dry nitrocellulose membrane, air-dried for 30 mins, blocked for one hour in blocking buffer, and then incubated for 1 hour at room temperature with streptavidin. Bead capture efficiency was measured using the formula of (pre-post)/pre x 100, once capture efficiency above 70% was achieved, the following steps were performed. **A-** Cropped blots showing the labeled dot blot for all the samples with their different replicates. **B-** The original blot.

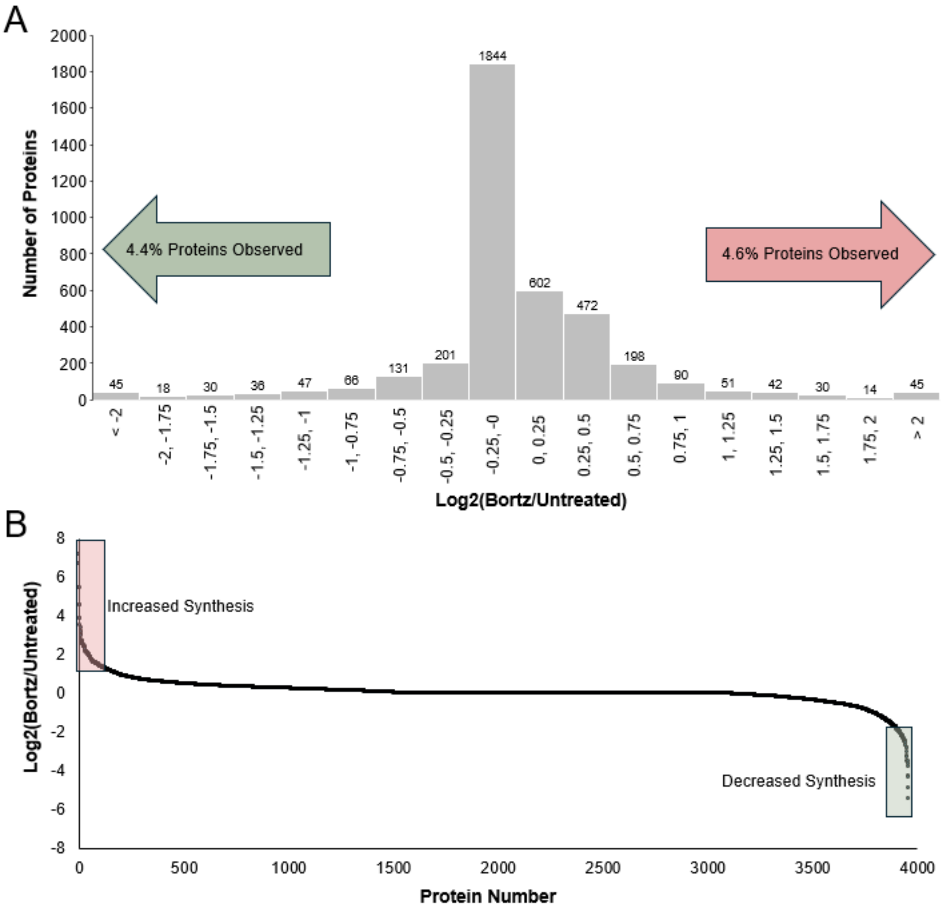

Figure S3: Analysis of Protein Synthesis Effect Size Distribution upon Bortezomib Treatment. (A) Histogram representing the frequency of occurrence for all observed log2(Fold Change) values in the Synthesis dataset. (B) Effect size distribution plot revealing all proteins ranked by their absolute value of their log2 (fold change).

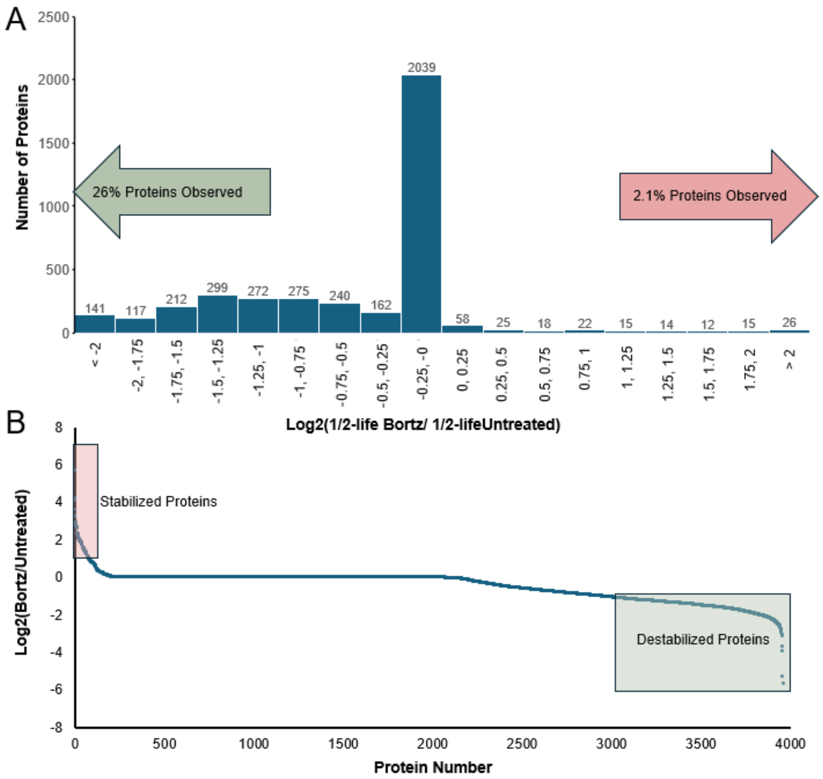

Figure S4: Analysis of Protein Degradation Effect Size Distribution upon Bortezomib Treatment. (A) Histogram representing the frequency of occurrence for all observed log2(Fold Change) values in the Degradation dataset. (B) Effect size distribution plot revealing all proteins ranked by their absolute value of their log2 (fold change).
